# Health-related physical fitness and blood pressure in people with intellectual disabilities in Korea

**DOI:** 10.1038/s41598-024-52039-x

**Published:** 2024-01-18

**Authors:** Bogja Jeoung, Do Young Pyun

**Affiliations:** 1 Department of Exercise Rehabilitation Welfare, College of Health Science, Gachon University, Incheon, Korea; 2 School of Sport, Exercise and Health Sciences, Loughborough University, Leicestershire, UK

**Keywords:** Health care, Population screening

## Abstract

Hypertension is one critical health issue causing cardiovascular diseases. There has been a common concern among health researchers that the prevalence of hypertension, known as high blood pressure, has been more frequently observed among people with intellectual disabilities, compared to the general population. Thus, this study aims to identify which health-related physical fitness and body composition factors are significantly related to both systolic and diastolic blood pressures among people with intellectual disabilities. The data from 2180 people with intellectual disability who conducted the physical fitness tests from 2019 to 2020 were used for this study. Four physical fitness tests (i.e., 3-min step, grip strength, sit-up, and sit and reach) and two body compositions (i.e., body mass index and body fat %) used as predictors, and two blood pressures (i.e., systolic blood pressure and diastolic blood pressure) were used as outcome variables. A regression analysis was performed to examine the proposed associations. The regression test revealed that 3-min step, body mass index, and body fat % were significantly associated with both systolic blood pressure and diastolic body pressure. This research contributes to our understanding of the roles of body compositions and aerobic endurance in preventing hypertension among people with intellectual disability.

## Introduction

Hypertension is one of the most significant problems in both medical and public healthcare because it is closely associated with mortality and serious diseases such as stroke, coronary artery disease, cardiac insufficiency, peripheral vascular disease, and renal failure^1^. Mortality related to ischaemic heart disease and stroke reportedly increases two-folds with each 20-mmHg increase in systolic blood pressure (SBP) or 10 mmHg increase in diastolic blood pressure (DBP)^2^. The significant and strong correlations between hypertension and cardiovascular diseases have been observed across different genders, ages, and ethnic groups, whereas the risk of cardiovascular diseases increases as blood pressure increases^3^. Hypertension is no less critical in people with intellectual disability (ID). The prevalence of cardiovascular diseases in people with ID has increased more rapidly than it has in the general population^4^. One previous study reported that mortality related to cardiovascular and respiratory diseases increased from the age of 40 years in people with ID^5^.

The hypertension guidelines and classification are not consistent across the western and eastern societies^6^. For example, the American College of Cardiology/American Heart Association defines hypertension as SBP ≥ 130 mmHg or DBP > 80 mmHg. However, the Korean Society of Hypertension^7^ considers this state as prehypertension and defines hypertension as SBP ≥ 140 mmHg or DBP > 90 mmHg, which is consistent with the cut-offs for hypertension recommended by the Japanese Society of Hypertension^8^.

Schroeder et al.^9^ reported that 48% of people with ID had hypertension stage 1 (SBP ≥ 130 mmHg, DBP > 80 mmHg) and 19.1% had hypertension stage 2 (SBP ≥ 140 mmHg, DBP ≥ 90 mmHg). Wyszynska et al.^10^ also reported that compared with the general population (15.5% prevalence of hypertension), people with ID in Poland showed 20.6% prevalence of hypertension. In another study, the prevalence of hypertension was higher in men than in women among people with ID, whereas it was higher in people with mild ID than it was in people with profound ID^11^. Likewise, Yang et al.^12^ reported that people with ID showed a higher prevalence of BP-related and cardiovascular diseases than did the general population in South Korea. The higher level of hypertension among people with ID seems due to a relatively low amount of and a low level of physical fitness^13^. To sum up, the past literature has evidenced a higher prevalence of hypertension among people with ID, compared to the general population. Thus, they would have more risks of developing various adult diseases^14^.

Among adult diseases, cardiorespiratory fitness is a powerful predictor that reduces the risks associated with metabolic syndrome, type II diabetes, and cardiovascular diseases^15^. It is also worth highlighting that cardiorespiratory fitness (i.e., “the ability of circulation and respiration to supply oxygen during sustained physical activity”) appears to be more effective in disease control, compared to physical activity (i.e., “voluntary movement produced by skeletal muscles that results in energy expenditure”)^16^. According to Williams^16^, physical fitness serves as a more robust risk factor in mitigating cardiovascular disease than mere physical activity, reporting that the risk decreased by approximately 25% with an increase in physical activity but by approximately 60% with an increase in physical fitness.

The Korea Paralympic Committee has developed the physical fitness test for people with disabilities to measure four fitness components: aerobic endurance, upper body muscle strength, muscle endurance, and flexibility^17^. Those components are assumed to reduce the likelihood of high blood pressure. One recent research^18^ confirmed that an individual’s physical fitness level was an important factor in preventing hypertension. For instance, people with high blood pressure had a 1.43 times higher risk of having lower grip strength^18^. Maintaining optimal physical fitness conditions is a crucial task in preventing high blood pressures among individuals. This study, accordingly, aims to examine how four physical fitness factors (i.e., 3-min step, grip strength, sit-up, and sit and reach) are associated with two types of blood pressure (i.e., SBP and DBP) in individuals with ID. There is also empirical evidence supporting a positive association between body compositions (i.e., body mass index [BMI] and waist circumference) and hypertension^18^. Additionally, BMI and body fat % are incorporated into the analysis as they are deemed useful measures for developing high blood pressures in this population^7^. As hypertension is diagnosed by either SBP or DBP, this study analyses them separately.

## Methods

### Research data

As a cross-sectional study, the data used for the analysis were drawn from the Incheon Fitness Standards Center of the Korea Paralympic Committee, which were publicly available through the Korea Culture Information Service Agency (https://www.bigdata-culture.kr/bigdata/user/data_market/detail.do?id=37c48c00-151f-11ec-bbc0-d7035fffebeb). This study included the participants with ID aged 13–50 years who lived in the region of Incheon, South Korea. The raw data of physical fitness tests from 2180 people with ID, who voluntarily participated at the centre from 2019 to 2020, along with their demographic information, were used in this study (see Table 1 for more demographic information of the participants). Informed consent was provided by the participants or their legal representatives. All methods were carried out in accordance with relevant guidelines and regulations.

**Table 1:** Characteristics of the participants (N = 2180).

	Total	Male	Female
Age (M ± SD)	27.58 ± 9.2	27.02 ± 9.0	28.5 ± 9.4
Gender	2180 (100%)	1360 (62.4%)	820 (37.6%)
Classification of disability*			
Level 1 (profound)	807 (37.0%)	503 (37.0%)	304 (37.1%)
Level 2 (moderate)	890 (40.8%)	537 (39.5%)	353 (43.0%)
Level 3 (mild)	428 (19.6%)	273 (20.1%)	155 (18.9%)
No response	55 (2.5%)	47 (3.5%)	8 (1.0%)
Height (cm)	162.3 ± 11.5	167.5 ± 9.5	153.7 ± 9.0
Weight (kg)	68.0 ± 18.8	72.04 ± 19.6	61.4 ± 15.4
BMI (kg/m^2^)	20.8 ± 5.0	21.3 ± 5.2	19.9 ± 4.5
Body fat %	31.7 ± 10.3	27.9 ± 9.6	37.1 ± 8.1
SBP (mmHg)	121.1 ± 14.9	122.7 ± 14.0	118.2 ± 15.9
DBP (mmHg)	77.6 ± 11.3	77.9 ± 10.9	77.05 ± 11.9
Aerobic endurance: 3-min step (PEI)	59.1 ± 6.4	57.6 ± 6.3	61.7 ± 5.8
Strength: grip strength (kg)	21.8 ± 8.3	24.4 ± 8.6	17.5 ± 5.6
Endurance strength: one minute sit-up (times)	20.5 ± 9.6	22.6 ± 9.9	15.9 ± 7.1
Flexibility: sit and reach (cm)	− 0.87 ± 13.0	− 2.8 ± 13.1	2.4 ± 12.1

*ID* intellectual disabilities, *BMI* body mass index, *SBP* systolic blood pressure, *DBP* diastolic blood pressure, *PEI* physical efficiency index.

*According to the Ministry of Health and Welfare (2013), ID is divided into three level, based on IQ scores: level 1 (IQ of less than 35); level 2 (IQ range of 35–49); and level 3 (IQ range of 50–70).

### Physical fitness measurement

The health-related physical fitness tests, which was developed for people with disabilities^17^, comprised six tests: (a) aerobic endurance (3-min step), (b) upper body muscle strength (hand grip strength), (c) muscle endurance (sit-up), (d) flexibility (sit and reach), and (e) body compositions (body mass index [BMI] and body fat %). BP. All physical fitness and body composition parameters measured at the Incheon Fitness Standard for People with Disabilities Center were used for this study. Each physical fitness test showed high internal consistency, with satisfactory reliability statistics (Cronbach’s alpha) ranging from 0.70 to 0.93^17^. All items were measured by four instructors with the national professional health and fitness certificates.

For physical fitness, firstly, aerobic endurance was measured via a 3-min step test. Using the step box (30-cm height for men and 20-cm height for women), the step test was performed at the beat of 30 steps per minute for men and 24 steps per minute for women. After the test, the participants were guided to take a seat, and their heart rates were measured for 30 s each across three different time points: between 1 and 1.5 min, between 2 and 2.5 min, and between 3 and 3.5 min. The measured heart rate was used to calculate physical efficiency index (PEI). For the participants aged 13–18 years, PEI was calculated with the equation ${\text{PEI}} = \frac{{{\text{D}}*100}}{{2*{\text{P}}}}$, and for women aged ≥ 18 years and men aged > 16 years, ${\text{PEI}} = 0.22*(300 - D) + \frac{{{\text{D}}*100}}{{5.5*{\text{P}}}}$, where ‘D’ indicates the step test duration (i.e., 180 s), and ‘P’ represents summed pulse counts across three points. Secondly, hand-grip strength test was used to measure the upper body muscle strength. This test was performed twice for the left and right hands using a hand dynamometer, and a higher value was recorded to the nearest 0.1 kg. Thirdly, sit-up test was used to estimate the endurance strength of the abdominal muscles and hip flexors. The participants were asked to touch their knees with elbows, return to the mat, and continue to perform as many as possible in 60 s. Lastly, sit and reach test was used to measure body flexibility. It measures the range of motion of the spine and hip during deep trunk flexion. The participants were instructed to sit with bare feet, legs extended, toes pointed up, and feet approximately hip-wide apart, with the soles of the feet against the base of the measuring device. They were then asked to push the slide slowly forward, as far as they could, by placing one hand on top of the other and without lifting their knees off the ground. Each participant performed the action twice, and a maximum height measurement was recorded to the nearest 0.1 cm.

For body compositions, BMI was calculated by dividing body weight (kg) by height in metre squared (m^2^). The percentage of body fat was measured by bioelectrical impedance analysis using a device called InBody 770 (InBody Co., Seoul, Korea). The participants’ BP values were also determined with an InBody BP-170. Before the BP measurements, the participants were guided to take a seat and have a 5–10 min rest before the BP-measuring device was placed on the left arm resting at the level of the heart. According to the Korean Society of Hypertension^7^, a normal BP category was defined as SBP < 120 mmHg and DBP < 80 mmHg, followed by elevated BP (120–129 mmHg for SBP and < 80 mmHg for DBP), prehypertension (130–139 mmHg for SBP or 80–89 mmHg for DBP), and hypertension (140 mmHg ≤ for SBP and 90 mmHg ≤ for DBP).

### Data analysis

The data were analysed using IBM SPSS 21.0. Firstly, descriptive statistics were calculated to present the means, standards deviation, frequencies, and percentages of the measures. In preparation for the regression analysis, the White test and Shapiro–Wilk test were employed to assess homoscedasticity and normality of residuals, respectively. Next, the variance inflation factor (VIF) was calculated to examine the multicollinearity among the independent variables (how much the variance of a regression coefficient is inflated due to multicollinearity in the equation). When a VIF of > 5.00^19^ is observed, this independent variable is removed from a regression analysis. Finally, a multiple linear regression was carried out to test the associations between the physical fitness tests and BPs. Age and gender were also included as control variables in the analysis. The significance level was set at the probability level of 0.05.

## Results

The demographic information and body compositions of the participants were measured and presented in Table 1. The total sample number was 2180, which comprised 1360 males (62.4%) and 820 females (37.6%). The average age was 27.6 ± 9.2, ranging from 13 to 50 years. The levels of classification of disability were as follows: 807 (37.0%) for level 1 (profound), 890 (40.8%) for level 2 (moderate), and 428 (19.6%) for level 3 (mild). For the further analyses, 1947 subjects who participated in the BP measurements were analysed. According to the BP standards, recommended by the Korean Society of Hypertension^7^, overall, 54.0% of the participants had normal BP, 12.9% had elevated BP, 20.7% had prehypertension, and 12.5% had hypertension. In terms of gender, 50.8% of the male and 59.7% of the female participants had normal BP, 14.4% of the male and 10.1% of the female participants had elevated BP, 22.8% of the male and 17.0% of the female participants had prehypertension, and 12.1% of the male and 13.3% of the female participants had hypertension (see Table 2). According to the descriptive statistics in Table 2, as age increased, the prevalence of normal BP decreased and that of hypertension increased. For the participants in age groups of 13–19 and 40–50, the prevalence of hypertension was slightly higher in male participants than in female participants, but it was slightly higher in female participants than in male participants for the age groups of 20–29 and 30–39. The prevalence of hypertension due to DBP was higher than that due to SBP.

**Table 2:** Prevalence of individuals with ID in the blood pressure categories (N = 1947).

	N	Normal Ave % (SBP %/DBP %)	Elevated Ave % (SBP %/DBP %)	Prehypertension Ave % (SBP %/DBP %)	Hypertension Ave % (SBP %/DBP %)
Overall	1947	54.0 (50.1/57.9)	12.9 (22.5/3.2)	20.7 (17.3/24.0)	12.5 (10.1/14.9)
Male	1226	50.8 (44.8/56.7)	14.4 (25.5/3.3)	22.8 (20.0/25.6)	12.1 (9.7/14.4)
Female	721	59.7 (59.2/60.1)	10.1 (17.3/2.9)	17.0 (12.8/21.2)	13.3 (10.7/15.8)
13–19	338	65.9 (58.9/72.9)	11.9 (22.2/1.5)	16.1 (13.3/18.9)	6.2 (5.6/6.8)
Male	252	63.1 (54.4/71.8)	12.1 (22.6/1.6)	18.3 (17.1/19.4)	6.6 (6.0/7.1)
Female	86	73.9 (72.1/75.6)	11.1 (20.9/1.2)	9.9 (2.3/17.4)	5.3 (4.7/5.8)
20–29	769	53.9 (50.5/57.2)	13.8 (23.9/3.6)	21.5 (16.5/26.4)	11.1 (9.1/13.0)
Male	469	49.4 (42.6/56.1)	16.7 (29.0/4.3)	23.2 (19.2/27.1)	10.9 (9.2/12.6)
Female	300	60.6 (62.7/58.5)	9.4 (16.0/2.7)	18.8 (12.3/25.2)	11.4 (9.0/13.7)
30–39	552	52.8 (49.8/55.8)	11.2 (19.4/2.9)	21.2 (19.2/23.2)	14.9 (11.6/18.1)
Male	347	50.6 (46.7/54.5)	11.7 (20.5/2.9)	23.5 (21.9/25.1)	14.3 (11.0/17.6)
Female	205	56.6 (55.1/58.0)	10.3 (17.6/2.9)	17.3 (14.6/20.0)	15.9 (12.7/19.0)
40–50	288	43.4 (39.6/47.2)	14.8 (25.0/4.5)	22.8 (20.5/25.0)	19.1 (14.9/23.3)
Male	158	35.4 (31.6/39.2)	17.7 (31.0/4.4)	27.6 (22.8/32.3)	19.4 (14.6/24.1)
Female	130	53.1 (49.2/56.9)	11.2 (17.7/4.6)	17.0 (17.7/16.2)	18.9 (15.4/22.3)

*ID* intellectual disabilities, *SBP* systolic blood pressure, *DBP* diastolic blood pressure.

Normal (< 120 for SBP and < 80 for DBP); Elevated (120–129 for SBP and < 80 for DBP); Prehypertension (130–139 for SBP or 80–89 for DBP); Hypertension (140 ≤ for SBP or 90 ≤ for DBP; Korean Hypotension Society, 2020).

In terms of assumption testing, first, the White test was used to detect heteroskedasticity of the residuals, revealing *χ*^2^(27) = 38.25, *p* = 0.074 for the SBP model and *χ*^2^(27) = 22.38, *p* = 0.718 for the DBP model. Both tests failed to reject the null hypothesis that there was homoscedasticity, and the residuals were not correlated with the value of the predictors, confirming the absence of heteroscedasticity issues. The Shapiro–Wilk test, however, did not yield statistical evidence supporting the normality of the residuals; *W* = 0.97, *p* < 0.001 for both unstandardised and standardised residuals. Lastly, the VIF was used as an indicator of multicollinearity as a prerequisite of the regression analysis. the VIF estimates of the independent variables ranged from 1.11 to 2.39, confirming no potential issues of multicollinearity in the model. The correlation coefficients ranging from − 0.26 to 0.59 supported the absence of multicollinearity.

Table 3 summarises the results of the regression analysis examining the effects of BMI, body fat %, 3-min step, grip strength, sit-up, and sit and reach test on both SBP and DBP at *p* = 0.05. In addition, age and gender were included in the analyses. Firstly, with regard to the regression of SBP, while all predictors, except sit and reach (*β* = − 0.02), showed significant associations with SBP at *p* = 0.05, grip strength (*β* = 0.20) and sit-up (*β* = 0.08) were positively related to SBP, which were unexpected, accounting for 20% of variance explained by the predictors. Next, regarding the regression of DBP, the test revealed that age (*β* = 0.22), BMI (*β* = 0.28), body fat % (*β* = 0.29), and 3-min step (*β* = − 0.13) were significantly related to DBP at *p* = 0.05, as expected. However, gender (*β* = − 0.04), sit-up (*β* = − 0.01) and sit and reach (*β* = − 0.04) were not significantly linked with DBP, and grip strength (*β* = 0.08) rather showed a positive association with DBP. The regression model explained 17% of variation in DBP that was predictable from the eight predictors.

**Table 3:** Linear regression analysis of BP and physical fitness factors (N = 1947).

	*β*	*t*	*p*	95% CI		*R*^2^
				Lower bound	Upper bound	
SBP						
Age	0.15	6.63*	< 0.001	0.17	0.32	0.20
Gender	− 0.15	− 6.47*	< 0.001	− 5.84	− 3.1	
BMI	0.33	15.40*	< 0.001	0.86	1.10	
Body fat %	0.28	10.98*	< 0.001	0.33	0.47	
3-min step	− 0.16	− 4.10*	< 0.001	− 0.56	− 0.20	
Grip strength	0.20	8.21*	< 0.001	0.27	0.44	
Sit-up	0.08	2.91*	< 0.01	0.04	0.20	
Sit and reach	− 0.02	− 0.96	0.337	− 0.08	0.03	
DBP						
Age	0.22	2.00*	0.049	0.001	0.05	0.17
Gender	− 0.04	− 1.77	0.076	− 1.98	0.10	
BMI	0.28	12.70*	< 0.001	0.53	0.72	
Body fat %	0.29	11.40*	< 0.001	0.26	0.37	
3-min step	− 0.13	− 3.25*	< 0.001	− 0.35	0.09	
Grip strength	0.08	3.22*	< 0.001	0.04	0.18	
Sit-up	− 0.01	− 0.53	0.598	− 0.05	0.08	
Sit and reach	− 0.04	− 1.77	0.076	− 0.07	0.004	

*BMI* body mass index, *SBP* systolic blood pressure, *DBP* diastolic blood pressure.

*Significant at *p* = 0.05.

## Discussion

To the best of our knowledge, this study is the first attempt to examine the prevalence of hypertension and the possible association between health-related physical fitness and hypertension among individuals with ID in South Korea. The overall prevalence of hypertension was 12.5%, and that of prehypertension was 20.7% among the selected population. Moreover, the prevalence of hypertension increased with increasing age. In this study, among participants aged 13–50 years, the mean prevalence rate of hypertension (SBP and DBP) was 19.1% in those aged 40–50 years, compared to 14.9% in those aged 30–39 years, 11.1% in those aged 20–29 years, and 6.2% in those aged 13–19 years. Moreover, the prevalence of hypertension due to DBP was higher than that due to SBP in all age groups.

While our findings are generally consistent with most of past studies conducted in different countries, there is a slightly different pattern in results between this and existing studies. For example, Schroeder et al.^9^ determined that the prevalence of hypertension was 19.1% and that of prehypertension was 28.9% in individuals with ID aged 18 years and over, which included athletes from across the globe who participated in the Special Olympics between 2014 and 2018. Moreover, according to age, the prevalence was 15.5% in individuals aged 18–29 years, 20.9% in those aged 30–39 years, and 25.9% in those aged 40–49 years, concluding that relatively a higher prevalence of prehypertension and hypertension was evidenced in Schroeder et al.^9^, compared to the current study. Moreover, the prevalence of hypertension in individuals with ID aged 15–18 years in Taiwan was 11.7%^11^, which was slightly higher than that in individuals aged 13–19 years in the present study (6.2%). However, for individuals aged 40–50 years, the prevalence of hypertension (19.1%) in people with ID in this study was slightly higher than that of Irish ID with a hypertension prevalence of 17.9% in individuals aged 44–49 years^20^. The prevalence of hypertension was also higher in male than in female participants in the study by O’Brein et al.^20^ but was higher in female than in male participants in the present study. Sari et al.^21^ reported a hypertension prevalence of 13.3% on SBP and 12.35% on DBP in people with ID (aged 19–49 years) in Turkey. They showed that the prevalence of hypertension was higher in male participants than in female participants, and those aged 14–18 years showed a higher hypertension prevalence rate on SBP (21.1%) than that on DBP (13.2%). To sum up, there were some extent of variations in the prevalence of hypertension across countries, age groups, and genders. This indicates that various environmental factors (e.g., diet, physical activity, and lifestyle) would be associated with BPs^22^. For example, the ‘Dietary Approaches to Stop Hypertension’ diet style, where people more consume fruit and vegetables but less consume meat, sweets, and alcohol, would lower SBP and DBP values^23^. Another example is related to smoking behaviour. People living in southeast Asia tend to show a higher smoking rate than those living in western countries^24^, and this behaviour would result in a higher prevalence of heart disease^25^. Such different sociocultural backgrounds may influence every country to adopt slightly different guidelines for hypertension. For example, while the American College of Cardiology^26^ defines hypertension stage 1 as SBP ≥ 130 mmHg or DBP ≥ 80 mmHg, the Korean Society of Hypertension^7^ considers this state as prehypertension and defined hypertension stage 1 as SBP ≥ 140 mmHg or DBP > 90 mmHg.

One common finding is that, compared with the general population, the incidence of hypertension is substantially higher in people with ID, and the prevalence of hypertension increases with an increase in age. According to the Korea Hypertension Fact Sheet 2019^27^, the prevalence rate of (pre)hypertension among general Korean adults over 30 years of age was 28%. Particularly, several past studies reported a significant associations between cardiopulmonary fitness factors and both SBP and DBP, thus emphasising the benefits of enhanced physical fitness^12,28, 29^. Some examples are, Kim et al.^29^ found enhancing cardiopulmonary fitness and muscular strength would prevent hypertension in people with ID. Kamil-Rosenberg^30^ identified low cardiopulmonary fitness and BMI > 25/m^2^ as factors that increased the prevalence of hypertension. The evidence from the past studies generally supports the current findings that body compositions and physical fitness are important for maintaining SBP and DBP levels. More specifically, health-related physical fitness comprises various components such as cardiopulmonary fitness, muscular strength and endurance, flexibility, and physical constitution, and, as noted, it is thus known to predict modifiable risk factors of chronic diseases with consequent effects on different health outcomes^31^. Thus, maintaining a good level of physical fitness is important for the prevention of chronic diseases in adulthood.

Possible reasons explaining the higher prevalence of hypertension among people with ID is lack of physical activities and a wide presence of obesity among people with ID, particularly older age groups^9^. Insufficient physical activity is highly likely to increase one’s vulnerability to overweight and obesity, which leads to a high incidence of chronic diseases and a consequent high probability of health deterioration and early death. Numerous studies have suggested guidelines on regular physical activity and nutrient requirements for the management of hypertension, with a notable report on the contribution of high-intensity physical activities and better nutrition in enhancing physical fitness^16,28, 32^. Interestingly, Williams^16^ reported that the hypertension prevalence could decrease by approximately 25% through an increase in physical activities but by approximately 60% through an increase in cardiorespiratory fitness, emphasising the importance of enhanced cardiorespiratory fitness. Additionally, So and Choi^33^ found a significant correlation between hypertension and physical fitness and suggested that an increase in physical fitness was essential in the prevention and treatment of hypertension.

It is important to implement a preventive programme for hypertension to promote healthy lifestyle for people with ID. For example, a long-term prevention programme including regular (e.g., annual) health screening, participation in a modest level of physical activities, consumptions of healthier foods and beverages, and maintenance of health-related fitness conditions can be provided to this population, particularly from their adolescent ages.

Unexpectedly, this study revealed that muscular strength was rather a positive indicator of blood pressures, which is inconsistent with the common finding from past literature^12,33, 34^. Possible reasons could be the average levels of muscular strength of people with ID are significantly lower than those of the general population, and their ranges (5–57 kg for grip strength) are relatively wide. These unexpected variations would explain why the study fails to show a negative association between grip strength and blood pressure. The inconsistent result calls for the need for continuous studies. Overall, two body composition (body mass fit and body fat %) and aerobic endurance are found as significant predictors in reducing hypertension among people in ID in Korea.

Lastly, several limitations surrounding this study should be noted. Firstly, as a cross-sectional research, the causes (body composition and physical fitness) and the effects (SBP and DBP) were measured at the same time. Thus, this study fails to show any causal relationships surrounding them. Future research may consider a longitudinal approach to examine the causal process, providing valuable information on the temporal order of the events underlying how the effects are determined across time and changes in body compositions and fitness conditions. Secondly, the data set used for this study did not provide information on physical activity levels (e.g., athletes or non-athletes) of the samples. A future study is designed to focus on a more homogeneous sample or compare across the different classification levels of physical activity to examine their interactions with body compositions/fitness conditions in explaining changes in blood pressures. Furthermore, it would be more logical to include a physical activity level as another predictor in the model, considering that all physical fitness measurements are related to physical activity behaviour. Unfortunately, due to the nature of the dataset, this research was unable to ascertain the specific contribution of physical activity within the samples. Investigating whether the effects of physical fitness on chronic disease remain independent from physical activity would be a worthwhile avenue for future research. Lastly, it should be noted that individuals with Down’s syndrome compose around 15–20% of the ID population and usually have some medial issues such as reduced muscle tone (hypotonia) and heart problems, likely resulting in lower grip strength and blood pressure, respectively^35^. While the data employed for this study did not provide additional information on Down’s syndrome, it is reasonable to assume that the sample include a significant number of people with Down’s syndrome which may have biased the current findings. For future research, it is suggested to recognise this population as a subgroup and analyse separately.

## Conclusions

The Korean government administers the national fitness test service aimed at promoting fitness and a healthy lifestyle among people with various disabilities. To cater to different disability groups, various fitness test batteries have been developed. For example, 3-min step and 5-min wheelchair run tests are used to measure aerobic endurance for people with ID and spinal cord disability, respectively. This study analysed the large secondary data set including fitness test results of individuals with ID in Korea. The investigation delved into the mechanisms elucidating how body compositions and physical fitness were associated with BP. It was evidenced that a notably high prevalence of hypertension among individuals with ID was associated with high levels of BMI and body fit % and a poor ability of 3-min step. Consequently, our findings contribute to the comprehending the role of body composition and aerobic endurance in preventing hypertension in this group. Accordingly, it is advised to implement diverse physical activity programmes designed to enhance aerobic performance, alongside the promotion of healthy lifestyles, such as nutritious dietary habits and regular exercise. This recommendation is particularly relevant for the prevention and management of hypertension, starting from the adolescent phase in individuals with ID.

## Data Availability

This study used secondary human data that have been released by the Korea Paralympic Committee and are publicly available through https://www.bigdata-culture.kr/bigdata/user/data_market/detail.do?id=37c48c00-151f-11ec-bbc0-d7035fffebeb.
